# The Effects of Schwann and Bone Marrow Stromal Stem Cells on Sciatic Nerve Injury in Rat: A Comparison of Functional Recovery

**Published:** 2012-06-13

**Authors:** Sam Zarbakhsh, Mehrdad Bakhtiyari, Abolfazl Faghihi, Mohammad Taghi Joghataei, Mehdi Mehdizadeh, Samideh Khoei, Korosh Mansouri, Behpour Yousefi, Vahid Pirhajati, Fatemeh Moradi, Maryam Shirmohammadi

**Affiliations:** 1. Department of Anatomy, Cellular and Molecular Research Center, Faculty of Medicine, Tehran University of Medical Sciences, Tehran, Iran; 2. Department of Anatomy, Faculty of Medicine, Semnan University of Medical Sciences, Semnan, Iran; 3. Department of Biophysics, Faculty of Medicine, Tehran University of Medical Sciences, Tehran, Iran; 4. Department of Physical Medicine and Rehabilitation, Tehran University of Medical Sciences, Tehran, Iran

**Keywords:** Bone Marrow Stromal Cells, Schwann Cells, Transplantation, Peripheral Nerve, Regeneration

## Abstract

**Objective:**

Transplantation of bone marrow stromal cells (BMSCs) or Schwann cells (SCs) can facilitate axonal regeneration in peripheral nerve injuries. The aim of this study was to compare the effects of transplantation of BMSCs and SCs on functional recovery after injury to the sciatic nerve in the rat.

**Materials and Methods:**

In this experimental research, adult male Wistar rats (n=24, 250-300 g) were used, BMSCs and SCs were cultured, and SCs were confirmed with anti S100 antibody. Rats were randomly divided into 3 groups (n=8 in each group): 1; control group: silicon tube filled with fibrin gel without the cells, 2; BMSCs group: silicon tube filled with fibrin gel seeded with BMSCs and 3; SCs group: silicon tube filled with fibrin gel seeded with SCs. The left sciatic nerve was exposed, a 10 mm segment removed, and a silicone tube interposed into this nerve gap. BMSCs and SCs were separately transplanted into the gap in the two experimental groups and were labeled with anti BrdU and DiI respectively. After 12 weeks electrophysiological and functional assessments were performed and analyzed by one-way analysis of variance (ANOVA).

**Results:**

Electrophysiological and functional assessments showed a significant difference between the experimental groups compared with the control group. Electrophysiological measures were significantly better in the SCs transplantation group compared with the BMSCs treatment group (p <0.05). Functional assessments showed no statistically significant difference between the BMSCs and SCs groups (p <0.05).

**Conclusion:**

Although both BMSCs and SCs have the potential to produce functional recovery after injury to the sciatic nerve in rats, electrophysiological evaluation confirms that the improvement after SCs transplantation is greater than that after BMSCs transplantation.

## Introduction

Peripheral nerve regeneration is an important clinical problem. The peripheral nerve system (PNS) has the potential to regenerate nerve cells, and peripheral nerve injury has been successfully repaired using various procedures such as nerve autograft, and nerve guidance tubes (1). In peripheral nerve injury one of the problems is suturing nerve ends when the resulting gap is too long (2). The nerve ends can be bridged with a nerve autograft to provide guidance for the regenerating nerves, but for a larger nerve trauma, a longer graft is required, and when the graft is thinner than the injured nerve, the transplantation of a bundle of nerve fibers is required. Because this procedure requires a large graft from a healthy nerve, sensory and motor destruction may occur at the donor site (3, 4).

 Axonal regeneration in a peripheral nerve injury needs extrinsic factors that promote growth, and supply guidance to the target. To overcome these problems, a variety of nerve guide tubes have been used to facilitate cell transplantation. The aims of cellular transplantation include: a. bridging the gap, b. providing a suitable environment to induce axonal regeneration, and c. promoting neovascularization. Different procedures have been applied to improve regeneration of peripheral nerves. One of those is the seeding of cells into the nerve guide tube (1, 4-6).

 Bone marrow stromal cells (BMSCs) have the ability to differentiate into different cell lines such as osteoblasts, cartilage, fat, ligament, muscle, and neurons (7, 8), and to contribute to the expression of many cytokines, and cellular factors (9, 10). It has been suggested that BMSCs implanted within nerve grafts synthesize neurotrophic factors and extracellular matrix components which play an important role in the survival and proliferation of axons, and Schwann cells (11). BMSCs can improve vascularization leading to injured tissue repair (6, 12). Thus BMSCs transplantation may result in recovery of the peripheral nerve after injury (11, 13).

Another cell type used for the repair of peripheral nerve injures is the Schwann cells (SCs). SCs and their basal lamina are crucial components in the environment through which regenerating axons grow to reach their peripheral targets. SCs can myelinate and produce physical support for axonal growth when they are injured (14). SCs of damaged peripheral nerves proliferate, help inflammatory infiltrating cells to remove debris, and up regulate trophic factors such as insulin-like growth factor (IGF), nerve growth factor (NGF), ciliary neurotrophic factor (CNTF), and laminin leading to axonal growth promoting. It can be inferred from this evidence that SCs transplantation may also promote recovery in the case of peripheral nerve injury (15, 16).

Our aim in this study was to compare the impact of both cell types (BMSC and SC) on peripheral nerve recovery as this has not been compared under similar conditions previously.

## Materials and Methods

### Animals

In this study, male Wistar rats (n=24, 250-300 g) were obtained from Pasteur Institute, Tehran, Iran. All animals had free access to laboratory chow, and tap water. Rats were randomly divided into 3 groups (n=8 in each group): 1. control group; 2. BMSCs transplantation group; 3. SCs transplantation group. All procedures in this study, including the use of animals, were approved by the Research Council of Tehran University of Medical Sciences (Tehran, Iran), Ethics Committee on Animal Experiments whose guidelines are in agreement with those of the National Institutes of Health for the use of live animals.

### Bone marrow stromal cells culture

Isolated preparation of BMSCs was performed according to the method described by Azizi et al. (17). Briefly rats were killed with an overdose of ketamin and femurs and tibias were dissected out. The marrow was then extruded with 10 ml of Dulbcco`s Modified Eagle Medium (DMEM) (Sigma Aldrich) and cultured in DMEM supplemented with 15% fetal bovine serum (FBS) (Sigma Aldrich), 2 mM L-glutamine (Sigma Aldrich), and 100 mg/ml kanamycine (Sigma Aldrich), incubated at 37℃, humidity 95%, and CO_2_ 5%. After 48 hours, the nonadherent cells were removed by replacing the medium. BMSCs were subcultured four times and finally used in the following experiments.

### Schwann cells culture

For the culture of SCs sciatic nerve was obtained under aseptic conditions. Rats were killed and their sciatic nerves were dissected bilaterally and placed at 4℃ into Hank`s buffered salt solution (HBSS) supplemented with 100 U/ml penicillin (Sigma Aldrich) and 100µg/ml streptomycin (Sigma Aldrich). The epineurium and connective tissue were removed under a microscope. The sciatic nerves were then cut into 2-3 mm fragments and dissociated using dispase (1.25 U/ml) (Sigma Aldrich) and collagenase (0.05%) (Sigma Aldrich) for 6-8 hours at 37℃. The tissue in this solution was triturated in a Pasteur pipette approximately ten times and was then incubated overnight at 37℃ in 5% CO_2_. The following day the explants were dissociated by gentle trituration. The mixture was then centrifuged at 103 rpm for 15 minutes at which point the supernatant was removed. The cells were resuspended in DMED medium containing 10% FBS, 2µM forskolin (Sigma Aldrich), 100 U/ml penicillin and 100 µg/ml streptomycin, and were placed in 35 mm petri dishes. Cells were incubated at 37℃ in 5% CO_2_, and the culture medium was changed every 48 hours. After 4 days in primary culture, cytosine arabinoside (Ara-C) (Sigma Aldrich) was added to the culture to a final concentration of 10-5 M, for a period of 3 days. This was replaced with DMEM containing 10% FBS, and 3 days later the Ara-C treatment was repeated. Cells were then maintained in DMEM with 10% FBS, 2mM L-glutamine, 100 U/ml penicillin, 100 µg/ml streptomycin, and 2 µM forskolin. After the cultures reached confluency, they were rinsed three times with phosphate buffered saline (PBS), and dissociated with 0.25% trypsin (Sigma Aldrich) and 1mM EDTA for 5 minutes at 37℃, and subcultured at a density of 5×10^3^ cells/cm^2^. The SCs were identified in living cultures on the basis of cell soma and nuclear morphology using phase contrast microscopy. In fixed culture, they were identified by immunocytochemical labeling for S100 protein (18).

### Transplantation procedure

Rats were anesthetized by intra peritoneal injection of a combination of ketamin (80 mg/kg), and xylazine (10 mg/kg). After skin incision the sciatic nerve was exposed using a muscle splitting incision. Under an operating microscope the left sciatic nerve was exposed at the mid-thigh, and a 10 mm segment of the nerve was severed and removed. A 12 mm silicone tube (1 mm inner diameter, 2 mm outer diameter) was interposed into this nerve gap (19). Both proximal and distal ends of the nerve were anchored into the conduit with 10-0 nylon suture. The silicone tube in the BMSCs group was filled with fibrin gel seeded with 500,000 BMSCs, while in the SCs group it was filled with fibrin gel seeded with 500,000 SCs, and the control group with fibrin gel without cells. Finally the skin was sutured with 5-0 silk.

### Immunohistochemistry of bone marrow stromal cells

BMSCs were labeled with a 3 µg/ml Bromodeoxyuridin (BrdU) (Sigma, Aldrich) solution added to the incubation medium 3 days prior to transplantation. After transplantation, the sections were incubated in 50% formamide (Merck, Germany), 2×SSC (Standard Sodium Citrate: 0.3M NaCl, and 0.03M Sodium Citrate) at 65℃ for 2 hours, washed for 10 minutes with 2×SSC at room temperature then incubated in 2M HCl (Merck, Germany) at 37℃ for 30 minutes. They were then rinsed in 0.1M Boric Acid (Merck, Germany) for 10 min, washed in PBS, and incubated with mouse anti-BrdU monoclonal antibody (Sigma Aldrich) at 4℃ overnight.

After rinsing 3 times in PBS for 10 minutes, the sections were incubated overnight in the dark at 4℃ with rhodamine conjugated secondary antibody (1: 100). They were then washed in PBS, covered with a coverslip, and were studied under fluorescence microscope (Olympus AX70) (20).

### Immunocytochemistry of Schwann cells

SCs were identified by immunocytochemical labeling for S100 protein. SCs were washed with 0.1 M PBS, fixed for 15 minutes in 4% paraformaldehyde, permeabilized for 30 minutes with 0.1 M PBS containing 0.3% Triton-X, and 1% normal goat serum, and incubated with rabbit anti-S100 antibody (Merck, Germany) for 60 minute at 37℃. After washes in PBS, SCs were incubated in secondary antibody, goat anti-rabbit cyanine 3.18-labelled IgG (1:200, Merck Germany), for 30 minute at room temperature. Following additional washes, SCs cultures were observed under a fluorescent microscope (Olympus AX70) (21).

### Histochemistry of Schwann cells

SCs were labeled using the fluorescent lipophilic tracer 1,1`-dioctadecyl-3,3,3`,3`-tetramethylindocarbocyanine perchlorate (DiI) (Sigma, Aldrich) prior to transplantation. For labeling, 1×106 cells/ml were resuspended in DMEM and 5µl/ml DiI was added. After incubation for 20 minuteat 37℃ with 5% humidified CO_2_, the cells were centrifuged for 5 minute and washed twice with PBS. They were then resuspended in PBS for transplantation. After transplantation frozen sections were prepared and the labeled cells were detected using fluorescent microscopy (Olympus AX70) (22).

### Electrophysiological study

Twelve weeks after transplantation, the rats were anesthetized intra peritoneally with ketamine (80 mg/kg) and xlylazine (10 mg/kg) and the sciatic nerves were exposed. Electric stimulation (duration of 0.1 ms, intensity of 2.3 mA) was applied to the proximal site of the injured nerve. The compound muscle action potential was recorded in the gastrocnemius with a needle electrode and a reference cap electrode inserted at the knee joint. The stainless steel needle used as the ground electrode was inserted into the tail skin. The area of the recorded muscle response (mV×ms), and the amplitude were calculated as these can be considered to reflect the amount of activated fibers (11, 23).

### Functional assessment

Functional evaluation of the sciatic nerve regeneration was expressed by the sciatic function index (SFI). Twelve weeks after surgery, the rats hind feet were dipped in ink and the rats were allowed to walk through a plastic tunnel so that the footprints could be recorded on paper loaded onto the bottom of the tunnel. The distance between the third toe ant heel (PL), first and fifth toe (TS), and second and fourth toe (ITS) was measured on the experimental side (EPL, ETS, and EITS, respectively), and the contra lateral normal side (NPL, NTS, and NITS, respectively). The SFI was calculated as follows: SFI=-38.3× (EPL-NPL)/NPL+109.5× (ETS-NTS)/NTS+13.3× (EITS-NITS)/NITS-8.8. In general, the SFI oscillates around 0 for normal nerve function, whereas around -100 SFI represents total dysfunction (23).

### Statistical analyses

All data were analyzed by one-way analysis of variance (ANOVA) followed by the Tukey test. Obtained data were presented as means ± standard deviation; and a level of p <0.05 was considered statistically significant.

## Results

### Bone marrow stromal cells culture

**Fig 1:**
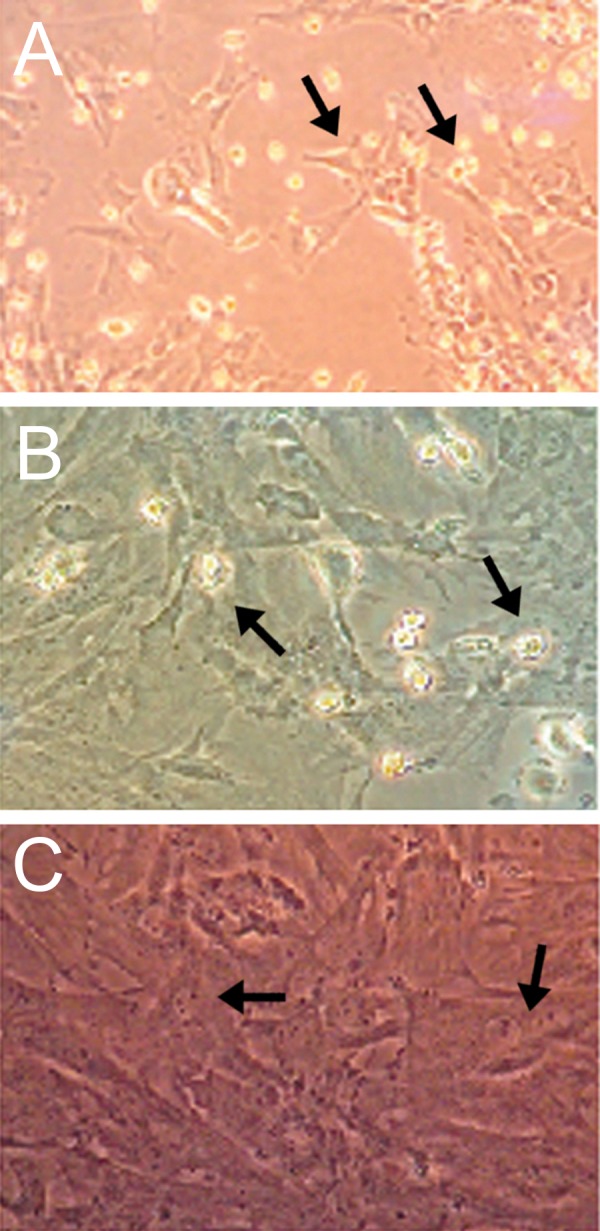
Cultured bone marrow stromal cells. (A) In P0 stage adherent cells exhibited small round, spindle-shaped (arrows) (×200). (B) In P2 stage. Most cells grew, and exhibited fibroblast-like morphology. The small rounded cells adhered to the surface of these cell layers (arrows) (×200). (C) In P4 stage rounded cells disappeared, and the fibroblast-like cells became morphologically homogeneous (arrows) (×200).

BMSCs obtained from the femurs and tibias of adult rats comprised heterogeneous groups of cells after seeding and growing in culture plates. After initial plating, the adherent cells exhibited either a small rounded-shape, a spindle-shape or a large flattened morphology (Fig 1A). Most cells grew and exhibited a fibroblast-like morphology on reaching confluence. The small rounded cells adhered to the surface of these cell layers (Fig 1B). These rounded cells disappeared after repeated passage, whereas the fibroblast-like cells became enriched. At passage 4 the fibroblast-like cells became morphologically homogeneous (Fig1C).

### Characterization of cultured Schwann cells

**Fig 2:**
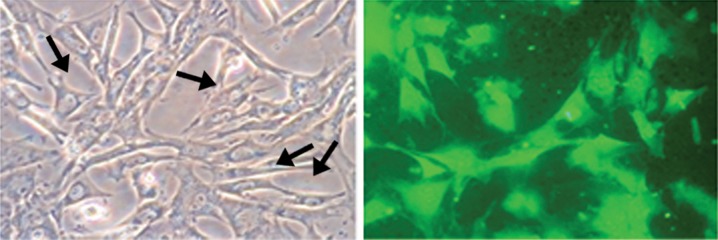
(A) Cultured Schwann cells were small, elongated, and spindle shaped in P2 stage (arrows) (×200). (B) Schwann cells were labeled with anti S100 antibody, and showed a tendency to line up side by side or end to end, and formed interconnected networks (arrow) (×200).

The spindle-shaped cellular morphology of the SCs seen on the culture plate was viable and there was no sign of infection. Most of the cells were small, elongated, and spindle shaped (Fig. 2A). Fluorescence microscopy showed the SCs were S100 positive cells. In the culture dishes the SCs had a tendency to line up side by side or end to end, and formed interconnected networks (Fig 2B).

**Fig 3:**
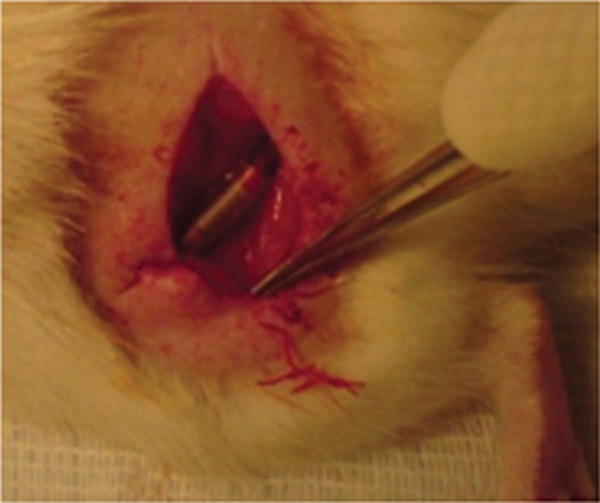
The left sciatic nerve was exposed at the mid-thigh, and a 10 mm segment of the nerve was removed. A 12 mm silicone tube was interposed into this nerve gap. Arrows show two heads of the nerve in the silicone tube.

### Finding from the immunohistochemistry of BMSCs and histochemistry of SCs

**Fig 4:**
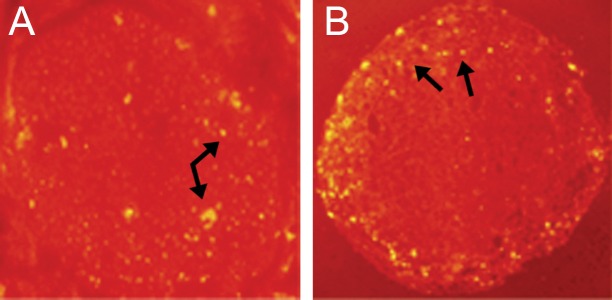
Cross-sections of the sciatic nerve, 4 weeks after transplantation of (A) BMSCs labeled with anti BrdU show as yellow points (arrows) (×100). (B) Schwann cells labeled with DiI show as yellow points around the nerve (arrows).

 Immunohistochemistry shows the BMSCs labeled with anti BrdU as yellow points in the cross section of sciatic nerve in Fig 4A, and histochemistry shows the SCs labeled with DiI as yellow points around the cross section of sciatic nerve in Fig 4B. Other tissues of the sciatic nerve cross sections are red (Fig 4 A, B). The results confirm the presence and viability of transplanted cells in the silicone tube bridging the gap 4 weeks after transplantation.

### Electrophysiology

**Fig 5:**
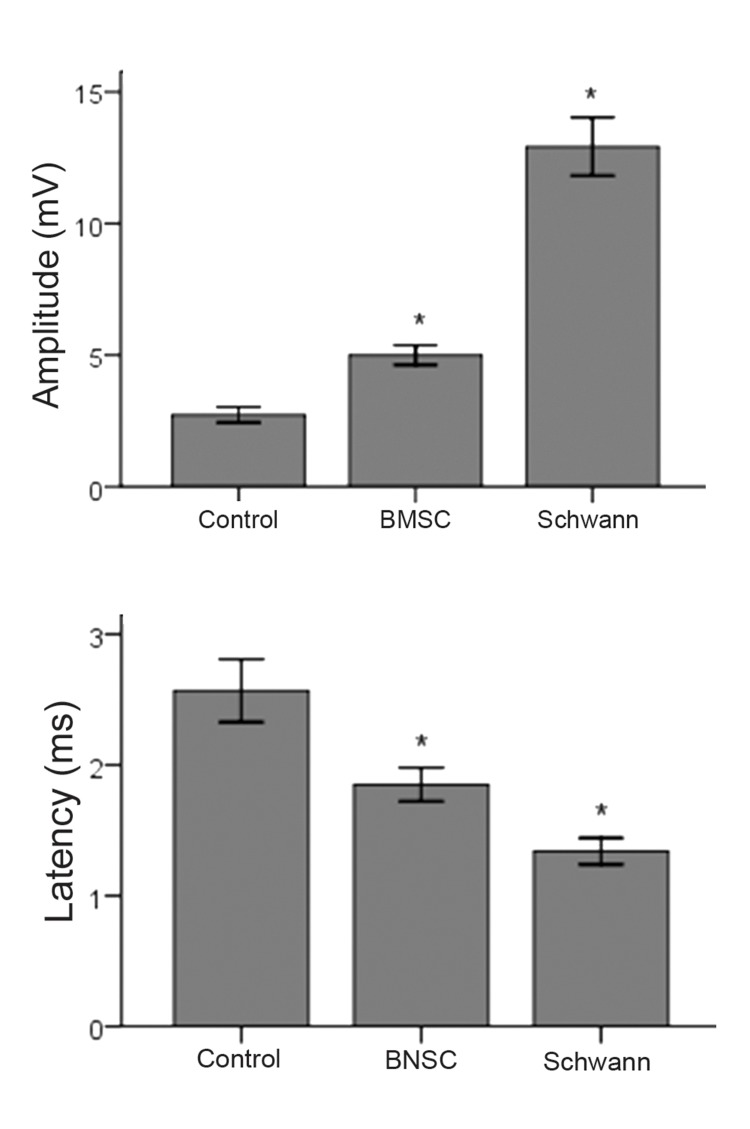
The results of electrophysiology tests of amplitude and latency showed there were statistically significant differences between the control group and the experimental groups (BMSCs and SCs)*; and there was a statistically significant difference between the BMSC group and SC group. *p <0.05.

**Fig 6:**
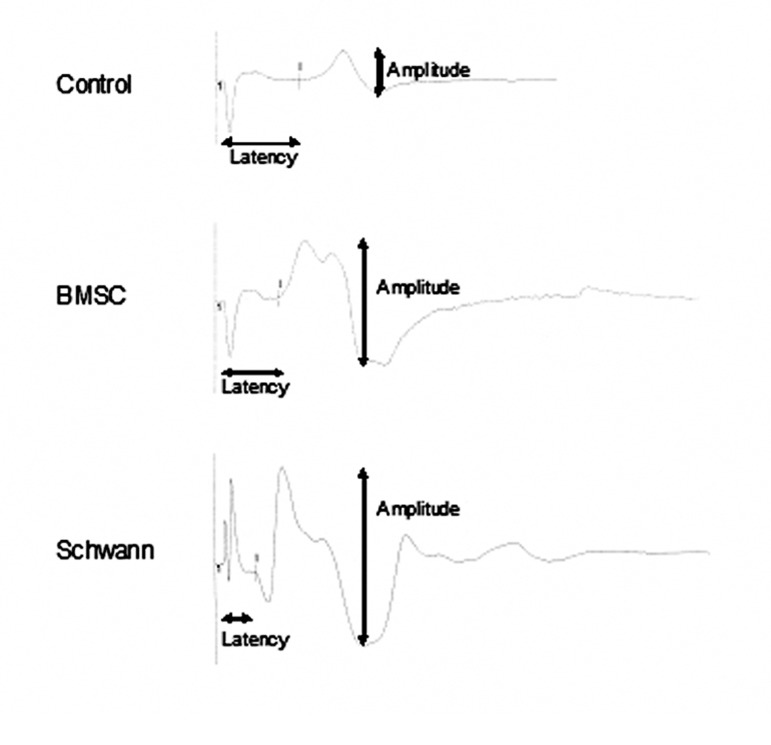
Electrophysiological waves for the control, BMSC and SC groups 12 weeks after surgery. Amplitude and latency is shown for each group. The time calibration bar was 2 ms and the amplitude calibration bar was 10 mV. The stimulation intensity was 2.3 mA and the duration was 0.1 ms.

Results of the electrophysiology tests comprise amplitude and latency. The mean amplitude in millivolts (mV) and the mean of latency in milliseconds is shown in figure 5. The time calibration bar was 2 ms and amplitude calibration bar was 10 mV. The stimulation intensity was 2.3 mA (milliampere) and the duration was 0.1 ms (Fig 6).

The results showed there were statistically significant differences between the control group and the experimental groups (BMSCs and SCs); and there was a statistically significant difference between BMSC group and SC group (p <0.05) (Fig 5).

### Functional analysis

**Fig 7:**
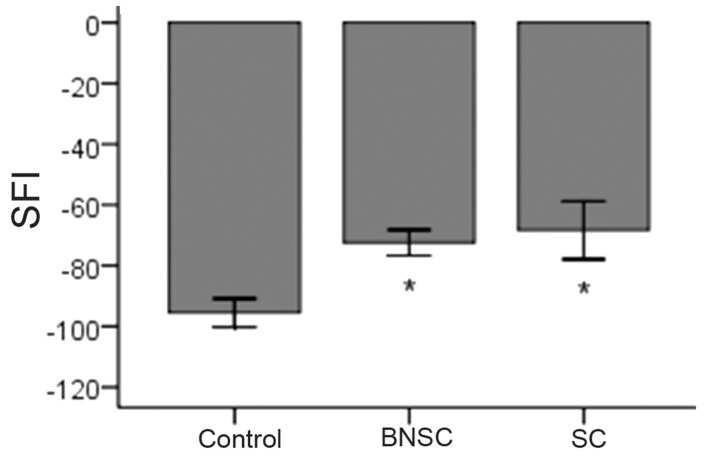
The results of the sciatic function index (SFI) test showed there were statistically significant differences between the control group and the experimental groups (BMSCs and SCs), but no statistically significant difference was observed between the BMSC group and SC group *p <0.05.

The results of SFI showed there were statistically significant differences between the control group and the experimental groups (BMSCs and SCs), but no statistically significant difference was observed between the BMSC group and SC group (p <0.05) (Fig 7).

**Fig 8:**
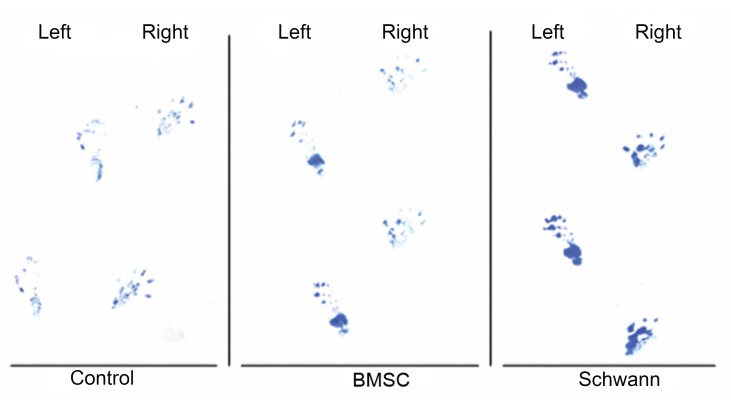
Foot print of control, BMSC and SC groups 12 weeks after surgery. Arrow shows walking direction.

## Discussion

Injuries of peripheral nerves, describe one of the most challenging microsurgical problems. These damages are associated with considerable disability due to loss of both motor and sensory functions. Development of a high quality replacement for auto grafts is needed because the auto graft procedure entails multiple surgeries, loss of function, and loss of sensation at the donor site (1, 4, 5).

In this study we compared the effect of transplantation of BMSCs and SCs on recovery of rat sciatic nerve injury. Several investigators have shown that BMSCs and SCs can repair injury to the sciatic nerve (11, 13, 15, 16). However, as they have not compared the results under similar conditions, their results are not comparable. Comparing these two methods under identical conditions may lead us to novel clinical approaches for utilizing these cells to replace peripheral nerve recovery auto grafts.

Because stem cells are important seeding cells for peripheral nerve regeneration, special attention has been given to the development of a rich and accessible cellular reservoir for this cell type (24). Several studies have showed the potential capability of using cultured BMSCs in peripheral nerve regeneration. BMSCs can promote axonal regeneration in PNS (11, 25). BMSCs can be aspirated directly from donors and include heterogenous populations of cells with distinct plasticity (24). During peripheral nerve regeneration trophic factors and supporting substances are essential molecules which play important roles, particularly for the regeneration of long nerve defects (19, 26). Researchers have indicated several mechanisms that may result in the promotion of functional improvement by BMSCs. These cells secrete many factors such as neurotrophic factors that induce tissue plasticity, and neuroprotective factors (27). BMSCs have the ability to release neurotrophic factors such as; NGF, BDNF, GDNF, CNTF and VEGF, as well as to produce extracellular matrix proteins such as; collagen I, collagen IV, fibronectin, and laminin (6, 11). Therefore there is good evidence to support the hypothesis that transplantation of BMSCs may repair peripheral nerve injuries (11, 13).

One of the effective cell candidates used in cell transplantation after peripheral nerve injuries are SCs. SCs are essential for the successful improvement of axonal degeneration. Peripheral nerve regeneration occurs mainly through a series of reactions produced by activated SCs so that the axons of the proximal nerve stump grow through the distal stump in close contact with the SCs. They produce various trophic factors for regenerating axons (28). Wallerian degeneration activates the SCs and they supply an ideal trophic milieu for axonal regeneration by secreting growth factors such as; NGF, HGF, VEGF and BDNF (29). In addition, SCs mediated by immunoglobulin superfamily molecules, like the nerve cell adhesion molecule, protein 0, cadherin, and the protocadherins, are important for axonal elongation and organized sprouting. SCs also produce basal lamina components, like collagen IV, and laminin that play an essential role in nerve regeneration. Among the adhesion molecules, laminin is the most potent factor for promoting axonal outgrowth. It thus seems likely that transplantation of SCs may also repair peripheral nerve injuries (3).

We cultured and labeled the BMSCs with BrdU. The immunohistochemistry process showed that BrdU-positive BMSCs can survive in the silicone tube at the gap after 4 weeks. The gap is located between the ends of the proximal and the distal of the nerve (Fig 3). This finding is consistence with previous reports (24, 30). The present study confirmed the presence of SCs by using anti S100 antibody *in vitro*, and confirmed the viability of the SCs by DiI staining in the silicone tube at the gap, after 4 weeks. This result also is in agreement with previous works (22, 30).

In this study, we showed the benefit of repairing 10 mm nerve defects by using BMSCs and SCs separately, as evidenced by electrophysiology tests in the gastrocnemius muscle, and walking behavior as measured by foot print analysis. The results of the electrophysiology tests showed there were statistically significant differences between the control group and the experimental groups (BMSCs and SCs). Some previously reported data support our findings (11, 21, 24, 31); Even though the findings of electrophysiology tests of the SCs transplanted group were significantly higher than the BMSCs transplanted group, the experiment had not been compared under similar conditions. The better recovery in the SC group was probably due to the direct and essential role of SCs in the regeneration and recreation of axonal bridges. The results of the SFI tests showed there were statistically significant differences between the control group and the experimental groups (BMSCs and SCs). These findings confirm those documented previously (11, 32 - 34); but the lack of any statistically significant difference observed between the BMSC group and SC group is new as these have not previously been compared under similar conditions.

As regards both cell types (BMSC and SC) have the ability to release neurotrophic factors such as; NGF, BDNF and VEGF, as well as to produce extracellular matrix proteins such as collagen IV and laminin (6, 11, 30), comparing them under similar conditions could be an important step in the selection of a repair procedure based on the measure of peripheral nerve recovery.

## Conclusion

Our results suggest that both BMSCs and SCs have the potential to generate functional recovery of injury to the sciatic nerve of the rat. Electrophysiological evaluation showed that the incorporation of SCs into nerve conduits produced better results than those of BMSCs. These findings provide greater support for use of SCs instead of BMSCs for the clinical repair of peripheral nerves damage.

## References

[1] Belkas JS, Shoichet MS, Midha R (2004). Peripheral nerve regeneration through guidance tubes. Neurol Res.

[2] Millesi H (1984). Nerve grafting. Clin Plast Surg.

[3] Ide C (1996). Peripheral nerve regeneration. Neurosci Res.

[4] Ishikawa N, Suzuki Y, Dezawa M, Kataoka K, Ohta M, Cho H (2009). Peripheral nerve regeneration by transplantation of BMSC-derived Schwann cells as chitosan gel sponge scaffolds. J Biomed Mater Res A.

[5] Dezawa M (2005). Future views and challenges to the peripheral nerve regeneration by cell based therapy. Rinsho Shinkeigaku.

[6] Fan W, Crawford R, Xiao Y (2011). The ratio of VEGF/PEDF expression in bone marrow mesenchymal stem cells regulates neovascularization. Differentiation.

[7] Sanches-Ramos J, Song S, Cardozo-Pelaez F, Hazzi C, Stedeford T, Willing A (2000). Adult bone marrow stromal cells differentiate into neural cells in vitro. Exp Neurol.

[8] Woodbury D, Schwarz EJ, Prockop DJ, Black IB (2000). Adult rat and human bone marrow stromal cells differentiate into neurons. J Neurosci Res.

[9] Dormady SP, Bashayan O, Dougherty R, Zhang XM, Basch RS (2001). Immortalized multipotential mesenchymal cells and the hematopoietic microenvironment. J Hematother Stem Cell Res.

[10] Bhagavati S, Xu W (2004). Isolationand enrichment of skeletal muscle progenitor cells from mouse bone marrow. Biochem Biophys Res Commun.

[11] Chen CJ, Ou YC, Liao SL, Chen WY, Chen SY, Wu CW (2007). Transplantation of bone marrow stromal cells for peripheral nerve repair. Exp Neurol.

[12] Marzban M, Bakhtiary M, Mehdizadeh M, Joghataei MT, Khoei S, PirhajatiMahabadi V (2010). Intravenous injection of human umbilical cord matrix stem cell (Wharton jelly stem cell) provides functional recovery in a rat model of traumatic brain injury. Cell Journal(Yakhteh).

[13] Braga-Silva J, Gehlen D, Roman JA, Menta C, Atkinson EA, Machado DC (2006). Bone marrow and platelet-rich plasma stem cells effects on nervous regeneration and functional recovery in an acute defect model of rats’ peripheral nerve. Acta Ortop Bras.

[14] Wakao S, Hayashi T, Kitada M, Kohama M, Matsue D, Teramoto N (2010). Long-term observation of auto-cell transplantation in non-human primate reveals safety and efficiency of bone marrow stromal cell-derived Schwann cells in peripheral nerve regeneration. Exp Neurol.

[15] Schlosshauer B, Muller E, Schroder B, Planck H, Muller HW (2003). Rat Schwann cells in bioresorbable nerve guides to promote and accelerate axonal regeneration. Brain Res.

[16] Lu MC, Chang YH, Chiang LC, Wang HT, Cheng CY, Yao CH (2006). Peripheral nerve regeneration through nerve guides filled with bilobalide, and Schwann cells. Biomed Eng Appl Basis Comm.

[17] Azizi SA, Stokes D, Augelli BJ, DiGirolamo C, Prockop DJ (1998). Engraftment and migration of human bone marrow stromal cells implanted in the brains of albino rats-- similarities to astrocyte grafts. Proc Natl Acad Sci U S A.

[18] Zurita M, Bonilla C, Otero L, Aguayo C, Vaquero J (2008). Neural transdifferentiation of bone marrow stromal cells obtained by chemical agents is a short-time reversible phenomenon. Neurosci Res.

[19] Chen YS, Hsieh CL, Tsai CC, Chen TH, Cheng WC, Hu CL (2000). Peripheral nerve regeneration using silicone rubber chambers filled with collagen, laminin and fibronectin. Biomaterials.

[20] Zurita M, Vaquero J (2006). Bone marrow stromal cells can achieve cure of chronic paraplegic rats: functional and morphological outcome one year after transplantation. Neurosci Lett.

[21] Rodriguez FJ, Verdu E, Ceballos D, Navarro X (2000). Nerve guides seeded with autologous Schwann cells improve nerve regeneration. Exp Neurol.

[22] Haastert K, Lipokatic E, Fischer M, Timmer M, Grothe C (2006). Differentially promoted peripheral nerve regeneration by grafted Schwann cells over-expressing different FGF-2 isoforms. Neurobiol Dis.

[23] Mimura T, Dezawa M, Kanno H, Sawada H, Yamamoto I (2004). Peripheral nerve regeneration by transplantation of bone marrow stromal cell-derived Schwann cells in adult rats. J Neurosurg.

[24] Wang D, Liu XL, Zhu JK, Jiang L, Hu J, Zhang Y (2008). Bridging small-gap peripheral nerve defects using acellular nerve allograft implanted with autologous bone marrow stromal cells in primates. Brain Res.

[25] Choi BH, Zhu SJ, Kim BY, Huh JY, Lee SH, Jung JH (2005). Transplantation of cultured bone marrow stromal cells to improve peripheral nerve regeneration. Int J Oral Maxillofac Surg.

[26] Lee AC, Yu VM, Lowe JB 3rd, Brenner MJ, Hunter DA, Mackinnon SE (2003). Controlled release of nerve growth factor enhances sciatic nerve regeneration. Exp Neurol.

[27] Song S, Kamath S, Mosquera D, Zigova T, Sanberg P, Vesely DL (2004). Expression of brain natriuretic peptid by human bone marrow stromal cells. Exp Neurol.

[28] Feneley MR, Fawcett JW, Keynes RJ (1991). The role of Schwann cells in the regeneration of peripheral nerve axons through muscle basal lamina grafts. Exp Neurol.

[29] Feng SQ, Zhou XF, Rush RA, Ferguson IA (2008). Graft of pre-injured sural nerve promotes regeneration of corticospinal tract, and functional recovery in rats with chronic spinal cord injury. Brain Res.

[30] Joghataei MT, Bakhtiari M, Pourheydar B, Mehdizadeh M, Faghihi A, Mehraein F (2010). Co-transplantation of Schwann and bone marrow stromal cells promotes locomotor recovery in the rat contusion model of spinal cord injury. Cell Journal(Yakhteh).

[31] Murakami T, Fujimoto Y, Yasunaga Y, Ishida O, Tanaka N, Ikuta Y (2003). Transplanted neuronal progenitor cells in a peripheral nerve gap promote nerve repair. Brain Res.

[32] Kim SM, Lee SK, Lee JH (2007). Peripheral nerve regeneration using a three dimensionally cultured Schwann cell conduit. J Craniofac Surg.

[33] Nie X, Zhang YJ, Tian WD, Jiang M, Dong R, Chen JW (2007). Improvement of peripheral nerve regeneration by a tissue-engineered nerve filled with ectomesenchymal stem cells. Int J Oral Maxillofac Surg.

[34] Hou SY, Zhang HY, Quan DP, Liu XL, Zhu JK (2006). Tissue-engineered peripheral nerve grafting by differentiated bone marrow stromal cells. Neuroscience.

